# Untangling the *Derogenes varicus* species complex in Scandinavian waters and the Arctic: description of *Derogenes abba* n. sp. (Trematoda, Derogenidae) from *Hippoglossoides platessoides* and new host records for *D. varicus* (Müller, 1784) *sensu stricto*[Fn FN1]

**DOI:** 10.1051/parasite/2024024

**Published:** 2024-05-22

**Authors:** Chahinez Bouguerche, Daniel C. Huston, Egil Karlsbakk, Mohammed Ahmed, Oleksandr Holovachov

**Affiliations:** 1 Department of Zoology, Swedish Museum of Natural History Box 50007 SE-104 05 Stockholm Sweden; 2 Australian National Insect Collection, National Research Collections Australia, CSIRO PO Box 1700 Canberra ACT 2601 Australia; 3 Department of Biological Sciences, University of Bergen PO Box 7803 N-5020 Bergen Norway; 4 Department of Evolution, Ecology and Behaviour, Institute of Infection, Veterinary and Ecological Sciences, University of Liverpool Liverpool L69 7AB UK

**Keywords:** *Derogenes varicus*, *Progonus muelleri*, cryptic species, *cox*1, Norway, Sweden

## Abstract

Several studies have shown that the euryxenic trematode *Derogenes varicus* (Müller, 1784) represents a species complex. Four lineages have been designated (DV1–4) with the DV1 clade corresponding to *D. varicus sensu stricto*. Herein, we investigate newly collected specimens of *D. varicus sensu lato* from Scandinavian and Arctic waters using integrative taxonomy. The trematodes were collected from *Melanogrammus aeglefinus*, *Eutrigla gurnardus*, *Trachinus draco*, and *Merluccius merluccius* off the Atlantic coast of Sweden and from *Hippoglossoides platessoides* from Arctic Svalbard. 28S sequences of derogenids from Sweden were identical to *D. varicus sensu stricto*, confirming its euryxeny. The 28S sequences of *Derogenes sp*. from *H. platessoides* were identical to *Derogenes* DV2 and differed from *D. varicus sensu stricto* by 3% and from *Derogenes* DV3 by 2%. The 28S sequence divergences of *Derogenes* sp. from *H. platessoides* with *D. ruber* and *D. lacustris* were 3 and 10%, respectively. ITS2 and *cox*1 divergences between *Derogenes* sp. from *H. platessoides* and other *Derogenes* species/lineages were at levels of interspecific differences. The species from *H. platessoides* is described here as *D. abba* n. sp. We also examined the type material of *Progonus muelleri* (Levinsen, 1881), the type and only species of the genus *Progonus*, with redescription and designations of paralectotypes. Based on specimens from Theodor Odhner’s collections at the Swedish Museum of Natural History, SMNH, Stockholm, we provide novel morphological and anatomical data for *D. varicus sensu lato* species complex. Lastly, we investigated Arthur Looss’s “lost collection” of Trematodes at the SMNH and characterised a putative species *Derogenes* sp. “*limula*”.

## Abstract (fr)

Plusieurs études ont montré que le trématode euryxene *Derogenes varicus* (Müller, 1784) représente un complexe d’espèces. Quatre lignées ont été désignées (DV1–4), le clade DV1 correspondant à *D. varicus sensu stricto*. Ici, nous étudions des spécimens nouvellement collectés de *D. varicus sensu lato* dans les eaux scandinaves et arctiques en utilisant la taxonomie intégrative. Les trématodes ont été collectés de *Melanogrammus aeglefinus*, *Eutrigla gurnardus*, *Trachinus draco* et *Merluccius merluccius* au large de la côte atlantique de la Suède et d’*Hippoglossoides platessoides* du Svalbard arctique. Les séquences 28S des Derogenidae de Suède étaient identiques à *D. varicus sensu stricto*, confirmant son euryxénie. Les séquences 28S de *Derogenes* sp. de *H. platessoides* étaient identiques à *Derogenes* DV2 et différaient de *D. varicus sensu stricto* par 3% et de *Derogenes* DV3 par 2%. Les divergences des séquence 28S de *Derogenes* sp. de *H. platessoides* avec *D. ruber* et *D. lacustris* étaient respectivement de 3 et 10%. Les divergences ITS2 et cox1 entre *Derogenes* sp. de *H. platessoides* et d’autres espèces/lignées de *Derogenes* se situaient à des niveaux de différences interspécifiques. L’espèce de *H. platessoides* est décrite ici comme *Derogenes abba* n. sp. Nous avons également examiné le matériel type de *Progonus muelleri* (Levinsen, 1881), type et seule espèce du genre *Progonus*, avec une redescription et des désignations de paralectotypes. Sur la base de spécimens des collections de Theodor Odhner au Musée suédois d’histoire naturelle (SMNH), Stockholm, nous fournissons de nouvelles données morphologiques et anatomiques sur le complexe d’espèces de *D. varicus sensu lato*. Enfin, nous avons étudié la « collection perdue » de Trématodes d’Arthur Looss au SMNH et caractérisé une espèce putative, *Derogenes* sp. « *limula* ».

## Introduction

A common problem in taxonomy and biodiversity assessment is the recognition of cryptic species. Although morphologically indistinguishable, cryptic species are genetically divergent, which may lead to unclear species boundaries between taxa. Additionally, the identification and study of cryptic species, along with the classification of organisms into nominal species, has importance beyond mere biodiversity assessment. This is particularly significant in the context of helminth parasites that impact human and veterinary health, as the presence of cryptic species can influence important medical and epidemiological factors such as pathogenicity and drug resistance [11].

The frequency of cryptic species varies among helminth groups [11]. Within the Trematoda, the richest known fauna of all the major metazoan taxa of fishes [6], cryptic species are frequently recognised with reports of them from at least 20 families [8]. Previous solid studies have demonstrated that encountering cryptic trematode species has become so frequent that their presence can almost be anticipated and should definitely be considered [12, 16]. In trematodes, firstly, there is the issue of phenotypic variation, which can mask common characteristics, potentially hindering identification. Secondly, there is the challenge of cryptic species, where species may appear similar externally but are genetically distinct [12]. Trematode species with similar morphology, but reported from wide host and geographic ranges, are often revealed as complexes with cryptic species [21]. Molecular techniques are indispensable for identifying cryptic species, as demonstrated in various trematode families [12]. The records of the derogenid *Derogenes varicus* (Müller, 1784), from 69 fish species across 33 families worldwide [3], displaying an exceptional euryxeny, hint that cryptic species may be present, and it has frequently been suggested that this species represents a species complex [19, 28]. Krupenko *et al*. [30] used molecular evidence to recognise four genetic lineages and infer that it is a species complex (designated as “DV1–4”). By analysing 28S sequences of *D. varicus* from the type-host, the Atlantic salmon *Salmo salar* from Norway, Bouguerche *et al*. [3] demonstrated that the DV1 clade is *D. varicus sensu stricto* (*s. s*.).

Herein, we investigate specimens of *D. varicus sensu lato* (*s. l*.) collected from several unrelated marine fish species, from Scandinavian and Arctic waters.

Based on DNA sequences of the second internal transcribed spacer (ITS2), the large subunit ribosomal DNA (28S) and the subunit I (*cox*1) in mtDNA and the examination of newly collected specimens, we provide a formal description of a new species in the genus *Derogenes* Lühe, 1900, recognised and described from the American plaice *Hippoglossoides platessoides*. We also identify *D. varicus s. s*. using 28S DNA sequences from additional host species. Examination of additional specimens of *D. varicus s. l*. from Sweden and Norway, Northeast Atlantic, from Theodor Odhner’s collections at the Swedish Museum of Natural History (SMNH), Stockholm, enabled us to provide novel morphological and anatomical data for *D. varicus s. l*. from various hosts. We also examined the type material of *Progonus muelleri* (Levinsen, 1881), the type and only species of the genus *Progonus* Looss, 1899 from the shorthorn sculpin *Myoxocephalus scorpius* from Aasiaat [Egedesminde], Greenland. Finally, we further investigated Arthur Looss’s “lost collection” of trematodes, bought by T. Odhner and preserved at the SMNH and we characterise a putative species *Derogenes* sp., an unpublished species referred to by A. Looss as “*Derogenes limula*”. The importance of integrative taxonomy is stressed here and demonstrated for species delimitations within the *D. varicus* species complex and in the genus *Derogenes*.

## Material and methods

### Host and parasite collection

Fishes were collected from off Sweden, Northeast Atlantic (Skagerrak, Kattegat, and Gullmarsfjorden) and off Svalbard, Arctic Norway (Table 1).

**Table 1:** Fishes examined from Scandinavian waters of the North Sea, Northeast Atlantic and the Arctic Ocean during this study.

Locality	Sweden, Northeast Atlantic		Norway, Arctic Ocean
Species	Gullmarsfjorden, Kristineberg, Skagerrak	Kattegat	Svalbard
*Eutrigla gurnardus*		31	
*Hippoglossoides platessoides*			5
*Melanogrammus aeglefinus*	8	14	
*Merluccius merluccius*		7	
*Trachinus draco*		29	

Specimens from Skagerrak and Kattegat were collected by the SLU Aqua team as part of the biannual International Bottom Trawl Survey, within the scope of their research projects and permits. Specimens from Gullmarsfjorden were collected in the vicinity of Kristineberg Center for Marine Research and Innovation, outside of the borders of the Gullmarns nature reserve, and within the scope of the permit for animal research from the Swedish Board of Agriculture (Enheten för försöksdjur och sällskapsdjur, Jordbruksverket, Dnr. 5.2.18-5483/18) and ethical approval for animal research from the Uppsala animal ethics committee (Uppsala djurförsöksetiska nämnd, Jordbruksverket, Dnr. 5.8.18-17209/2021) issued to the Swedish Museum of Natural History. Specimens from the Arctic Ocean were collected during the HHUMTL22 cruise by the Arctic University Museum of Norway, within the scope of the fieldwork sampling permit issued by the governor of Svalbard (RiS-1D12021Al) and the permission to trawl from the Norwegian Directorate of Fisheries (21/16250). Fishes were euthanised and made available for examination.

Several specimens of greater weever *Trachinus draco*, grey gurnard *Eutrigla gurnardus*, European hake *Merluccius merluccius*, and haddock *Melanogrammus aeglefinus* from Skagerrak and Kattegat were collected during the biannual International Bottom Trawl Survey by the SLU along the Swedish coast (Table 1). Eight specimens of *M. aeglefinus* from Gullmarsfjorden were collected in the vicinity of the Kristineberg Center for Marine Research and Innovation.

The American plaice *H. platessoides* from the Arctic Ocean was collected by bottom trawl, at 79 59.860558 N, 15 27.879328 E, and 170 m depth [68].

Digeneans were collected from freshly killed fishes. The gastrointestinal tract was removed and examined for trematodes using the gut wash method [14, 26]. Digeneans were fixed in near-boiled saline without pressure and preserved immediately in 80% ethanol for morphological and molecular studies. Nine specimens were processed as hologenophores (sensu Pleijel *et al*. [53]). Type specimens of *P. muelleri* were requested from the Natural History Museum of Denmark (SNM) and nine specimens of *P. muelleri* from *M. scorpius* from Aasiaat [Egedesminde] Greenland preserved in 70% ethanol and marked as holotype (old catalogue number ZMUC-TRE-000032) were received. Two of the specimens were stained in acetocarmine and studied by microscopy.

Additional studied specimens include derogenids in the invertebrate collections at the Swedish Museum of Natural History (SMNH): 1. *D. varicus s. l*. from the Northeast Atlantic from T. Odhner’s collections, ex the European flounder *Platichthys flesus* from Gullmarsfjorden, Kristineberg, Sweden, the argentina *Argentina sphyraena*, the common ling *Molva molva*, the cusk *Brosme brosme*, and the Atlantic halibut *Hippoglossus hippoglossus* from Trondheim, Norway; 2. *P. muelleri* ex *M. scorpius* from Spitsbergen, Svalbard, Arctic Norway; 3. *Derogenes* sp. ex the tentacled blenny *Parablennius tentacularis* from Trieste, Italy, Central Mediterranean from A. Looss’s collection.

### Morphological methods

Preserved specimens of *Derogenes* including hologenophores were stained in iron acetocarmine, destained with acid-alcohol (1% HCl in 70% ethanol), dehydrated in an ethanol series (70–100%), cleared in clove oil, and mounted in Canada balsam. Two specimens of the type material of *P. muelleri* were stained according to the same methods and mounted on two separate slides (NHMD-114950).

Drawings were made through a Nikon Eclipse i80 microscope with DIC (differential interference contrast) and a drawing tube. Drawings were scanned and redrawn on a computer with Adobe Illustrator 2023. Stained specimens were measured by ImageJ ver. 1.53K [57]. Measurements are in micrometres and indicated as the range followed by the number of measurements in parentheses. Types and vouchers were deposited at the Swedish Museum of Natural History (SMNH), Stockholm, Sweden; the Natural History Museum of Denmark (SNM), Copenhagen, Denmark and the Arctic University Museum of Norway (UiT), Tromsø, Norway.

### Molecular methods

Genomic DNA was extracted from seven hologenophores of *D. varicus s. l*. from *T. draco*, *E. gurnardus*, *M. merluccius*, *M. aeglefinus*, and two hologenophores of *Derogenes* sp. from *H. platessoides*. Genetic data were generated for three markers: *cox*1, ITS2, and the 28S rDNA. Small fragments of each hologenophore (posterior third) were placed in a 1.5 mL microcentrifuge tube containing 20 μL buffer ATL (QIAGEN, Hilden, Germany). For extraction of genomic DNA (gDNA), 20 μL buffer ATL and 20 μL proteinase K were added to each sample, followed by vortexing and incubation in an incubating microplate shaker at 56 °C and 300 rpm overnight. The lysed samples were processed to obtain gDNA, following the manufacturer’s instructions for gDNA extraction using a QIAGEN QiAmp DNA Microkit. The PCR reaction was performed following Bouguerche *et al*. [3].

The *cox*1 was amplified with the primers and cycling profile used by Krupenko *et al*. [30]. Primers, amplification, and sequencing protocols for the 28S rDNA regions followed Pérez-Ponce de León and Nadler [52] and García-Varela and Nadler [17]. ITS2 sequences were amplified using the primers 3S [45] and ITS2.2 [13]. PCR products were purified by Ampure XP Kit (Beckman Coulter Inc., Brea, CA, USA) and sequenced in both directions on a 3730 l DNA Analyser 96-capillary sequencer (Applied Biosystems, Waltham, MA, USA). We used CodonCode Aligner 3.7.1 (Codon Code Corporation, Centerville, MA, USA) to edit sequences, compared them to the GenBank database content with BLAST, and deposited them in GenBank under accession numbers PP314018, PP314019, PP314020, PP314022, PP384389, and PP384390.

### Trees and distances

Phylogenetic analyses were performed using the newly generated sequences of *Derogenes* spp. and those of related species available in GenBank (Table 2), mainly the *D. varicus* complex and *P. muelleri* complex provided by Krupenko *et al*. [30] and by Bouguerche *et al*. [3]. Alignments for each gene region were constructed separately in AliView [32], then trimmed to the shortest sequence. Phylogenetic tree inference was carried out by the maximum likelihood (ML) method using MEGA11 [66]. Nucleotide substitution models for phylogenetic analyses using the ML method were selected using MEGA11 [66]. The Hasegawa-Kishino-Yano with Gamma Distributed (HKY+ G) model [23] was selected for the 28S, the Kimura 2-parameter (K2) model [27] for ITS2, and the Tamura-Nei model with Gamma Distributed with Invariant sites (TN93+ G+I) [65] for *cox*1. The probabilities were computed by the bootstrap analysis of 500 replications. We also constructed phylogenetic trees of respective regions for the same data sets using the neighbour-joining (NJ) method [56] with MEGA11, with 2,000 bootstraps computed for *cox*1, ITS2, and 28S. The *p*-distances [27] were computed from the same datasets with MEGA11.

**Table 2:** Collection data for sequences analysed in this study. ^a^Two sequences by Krupenko *et al*. [30] are wrongly annotated on GenBank: OM761977 and OM762017 and these two *Derogenes varicus* complex sp. DV1 isolates are in fact DV2.

Species	Host	Location	GenBank ID			Source
			28S	ITS2	*Cox*1	
*D. varicus s.s.*	*Melanogrammus aeglefinus*	Skagerrak, Sweden, NEA	PP297456	–	–	Present study
*D. varicus s.s.*	*Melanogrammus aeglefinus*	Skagerrak, Sweden, NEA	PP297457	–	–	Present study
*D. varicus s.s.*	*Melanogrammus aeglefinus*	Kattegat, Sweden, NEA	PP297459	–	–	Present study
*D. varicus s.s.*	*Eutrigla gurnardi*	Kattegat, Sweden, NEA	PP297458	–	–	Present study
*D. varicus s.s.*	*Eutrigla gurnardi*	Kattegat, Sweden, NEA	PP297460	–	–	Present study
*D. varicus s.s.*	*Trachinus draco*	Kattegat, Sweden, NEA	PP297461	–	–	Present study
*D. varicus s.s.*	*Merluccius merluccius*	Kattegat, Sweden, NEA	PP297462	–	–	Present study
*D. varicus s.s.*	*Merluccius merluccius*	Kattegat, Sweden, NEA	PP297463	–	–	Present study
*Derogenes abba* n. sp.	*Hippoglossoides platessoides*	Svalbard, AO	PP314018	PP314020	PP384389	Present study
*Derogenes abba* n. sp.	*Hippoglossoides platessoides*	Svalbard, AO	PP314019	PP314022	PP384390	Present study
*D. varicus s.s.*	*Gadus morhua*	Sweden, NEA	OQ916447	OQ916446	OR140896	[3]
*D. varicus s.s.*	*Gadus morhua*	Norway, AO	OQ916454	OQ916451	OR140779	[3]
*D. varicus s.s.*	*Gadus morhua*	Norway, AO	OQ916441	OQ916449	OR140895	[3]
*D. varicus s.s.*	*Gadus morhua*	Norway, AO	OQ916448	OQ916439	OR140832	[3]
*D. varicus s.s.*	*Merlangius merlangus*	Sweden, NEA	OQ916442	OQ916456	OR507183	[3]
*D. varicus s.s.*	*Merlangius merlangus*	Sweden, NEA	OQ916445	OQ916457	OR507185	[3]
*D. varicus s.s.*	*Merlangius merlangus*	Sweden, NEA	OQ916444	OQ916443	OR140909	[3]
*D. varicus s.s.*	*Merlangius merlangus*	Sweden, NEA	OQ916437	OQ916452	OR140894	[3]
*D. varicus s.s.*	*Merlangius merlangus*	Sweden, NEA	OQ916450	OQ916438	OR140897	[3]
*D. varicus s.s.*	*Merlangius merlangus*	Sweden, NEA	OQ916440	OQ916453	OR507184	[3]
*D. varicus s.s.*	*Salmo salar*	Norway, NEA	OQ916455	–	–	[3]
*D. varicus s.s.*	*Gadus morhua*	Russia, WS	OM761963	OM762003	OM807174	[30]
*D. varicus s.s.*	*Myoxocephalus scorpius*	Russia, WS	OM761964	OM762004	OM807175	[30]
*D. varicus s.s.*	*Anarhichas lupus*	Russia, WS	OM761965	OM762005	OM807176	[30]
*D. varicus s.s.*	*Limanda limanda*	Russia, WS	OM761966	OM762006	OM807177	[30]
*D. varicus s.s.*	*Eleginus nawaga*	Russia, WS	OM761967	OM762007	OM807178	[30]
*D. varicus s.s.*	*Limanda limanda*	Russia, WS	OM761968	OM762008	OM807179	[30]
*D. varicus s.s.*	*Limanda limanda*	Russia, WS	OM761962	OM762002	OM807173	[30]
*D. varicus s.s.*	*Clupea pallasii*	Russia, WS	OM761969	OM762009	OM807180	[30]
*D. varicus s.s.*	*Clupea pallasii*	Russia, WS	OM761970	OM762010	OM807181	[30]
*D. varicus s.s.*	*Triglops murrayi*	Russia, WS	OM761976	OM762016	–	[30]
*D. varicus s.s.*	*Gadus morhua*	Russia, BS	OM761971	–	OM807182	[30]
*D. varicus s.s.*	*Myoxocephalus scorpius*	Russia, BS	OM761972	OM762012	OM807183	[30]
*D. varicus s.s.*	*Myoxocephalus scorpius*	Russia, BS	OM761973	OM762013	OM807184	[30]
*D. varicus s.s.*	*Gadus morhua*	Russia, WS	OM761974	OM762014	–	[30]
*D. varicus s.s.*	*Gadus morhua*	Russia, WS	OM761975	OM762015	–	[30]
*D. varicus s.s.*	*Cryptonatica affinis*	Russia, WS		OM762024	–	[30]
*D. abba* n. sp.	*Buccinum scalariforme*	Russia, WS	OM761977 ^a^	OM762017 ^a^	–	[30]
*D. abba* n. sp.	*Amauropsis islandica*	Russia, WS	OM761989	OM762029	–	[30]
*D. abba* n. sp.	*Euspira pallida*	Russia, WS		OM762030	OM807194	[30]
*D. abba* n. sp.	*Euspira pallida*	Russia, BS		OM762031	OM807195	[30]
*D. abba* n. sp.	*H. platessoides*	UK, NEA	AY222189			[50]
*D. varicus* DV3	*Eumicrotremus fedorovi*	North Pacific	MW504598	–	–	[63]
*D. varicus* DV3	*Eumicrotremus fedorovi*	North Pacific	MW504599	–	–	[63]
*D. lacustris*	*Oncorhynchus mykiss*	Lake Gutiérrez, Argentina	–	–	LC586095	[67]
*D. lacustris*	*Salvelinus fontinalis*	Lake Gutiérrez Argentina	–	–	LC586094	[67]
*D. lacustris*	*Percichthys trucha*	Lake Morenito, Argentina	–	–	LC586093	[67]
*D. lacustris*	*Percichthys trucha*	Lake Nahuel Huapi Argentina			LC586096	[67]
*D. lacustris*	*Galaxias maculatus*	Lake Gutiérrez, Argentina	–	–	LC586092, LC586097, LC586098	[67]
*D. lacustris*	*Galaxias maculatus*	Lake Escondido, Argentina	LC586089	–	–	[67]
*D. lacustris*	*Galaxias maculatus*	Lake Gutiérrez, Argentina	LC586090	–	–	[67]
*D. ruber*	*Chelidonichthys lucerna*	Algeria, WM	OQ919799	OQ919806	OR245386	[18]
*D. ruber*	*Chelidonichthys lucerna*	Algeria, WM	–	OQ919798	OR245546	[18]
*D. ruber*	*Chelidonichthys lucerna*	Algeria, WM	–	OQ919804	–	[18]
*D. ruber*	*Chelidonichthys lucerna*	Algeria, WM	OQ919800	OQ919801	OQ919800	[18]
*D. ruber*	*Chelidonichthys lucerna*	Algeria, WM	OQ919803	OQ919802	–	[18]
*P. muelleri*	*Eumicrotremus fedorovi*	North Pacific	MW507469	–	–	[63]
*P. muelleri*	*Eumicrotremus fedorovi*	North Pacific	MW507470	–	–	[63]
*P. muelleri*	*Caprella septentrionalis*	North Pacific	MW507471	–	–	[63]
*P. muelleri* PM2	*Myoxocephalus scorpius*	Russia, WS	OM761978	OM762018	OM807185	[30]
*P. muelleri* PM1	*Myoxocephalus scorpius*	Russia, BS	–	–		[30]
*P. muelleri* PM1	*Myoxocephalus scorpius*	Russia, WS	OM761979	OM762019	OM807186	[30]
*P. muelleri* PM1	*Myoxocephalus scorpius*	Russia, WS	OM761980	OM762020	–	[30]
*P. muelleri* PM1	*Myoxocephalus scorpius*	Russia, WS	OM761981	OM762021	–	[30]
*P. muelleri* PM1	*Limanda limanda*	Russia, WS	OM761982	OM762022	–	[30]
*P. muelleri* PM1	*Triglops murrayi*	Russia, WS	OM761983	OM762023	–	[30]
*Allogenarchopsis problematica*	*Semisulcosipra reiniana*	Japan, NWP	MH628313	–	–	[64]
*Genarchopsis chubuensis*	*Rhinogobius flumineus*	Japan, NWP	MH628311	–	–	[64]
*Genarchella* sp. 1	*Herichthys labridens*	San Luis Potosí Mexico	MK648276	–	–	[15]
*Genarchella* sp. 1	*Astyanax aeneus*	Yucatán, Mexico, WCA	MK648277	–	–	[15]
*Thometrema lotzi*	*Lepomis microlophus*	Pascagoula River, USA	KC985236	–	–	[10]
*Thometrema patagonica*	*Percichthys trucha*	Lake Pellegrini, Argentina	LC586091	–	–	[67]
*Prosogonotrema bilabiatum*	*Caesio cuning*	Heron Island, Australia	AY222191	–	–	[50]
*Accacladocoelium macrocotyle*	*Mola mola*	Spain, WM	–	KF687303	–	[1]
*Didymocystis wedli*	*Thunnus orientalis*	Japan, NWP	–	–	AB725624	Unp.

*S. s*., *sensu stricto*. AO, Arctic Ocean. BS, Barents Sea. NEA, Northeast Atlantic. NWP, Northwest Pacific. WCA, Western-Central Atlantic. WM, Western Mediterranean. WS, White Sea.

## Results

### Morphology

Family Derogenidae Nicoll, 1910

Subfamily Derogeninae Nicoll, 1910

Genus *Derogenes* Lühe, 1900

#### *Derogenes abba* n. sp. (Figs. 1A–1C)

urn:lsid:zoobank.org:act:7CB281BD-05E0-4BAF-A717-A1E4E1477AE1

**Figure 1:**
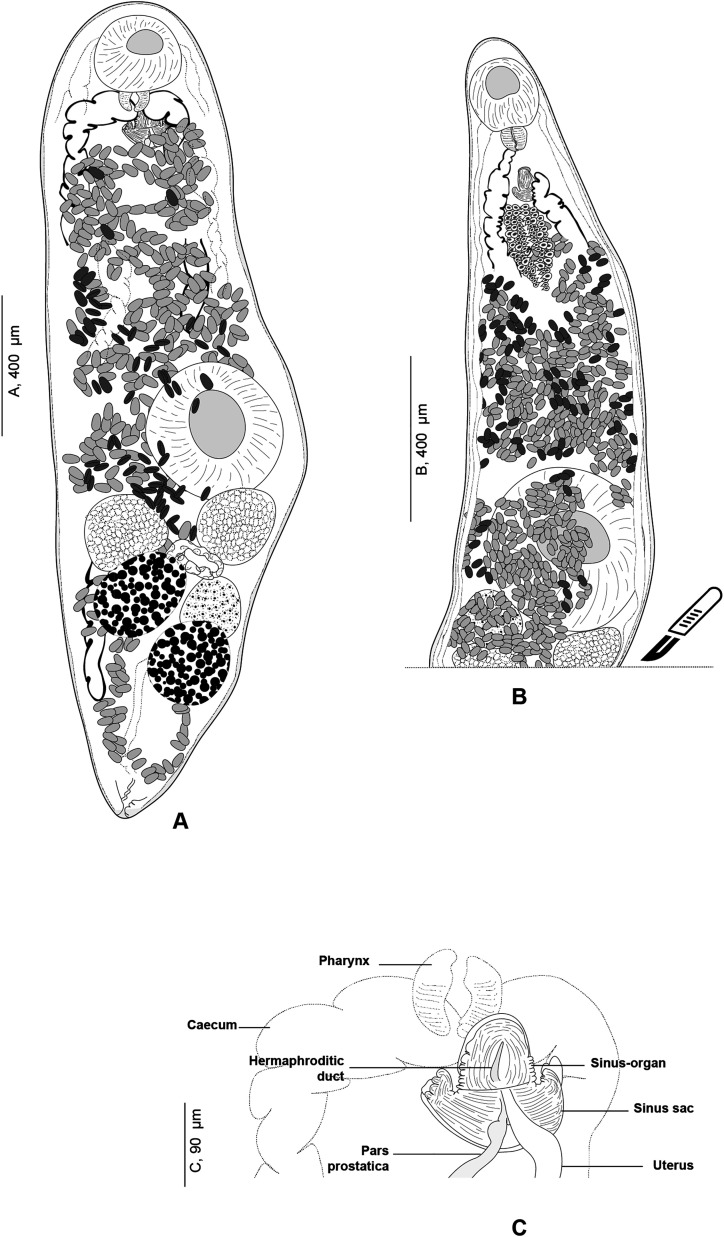
*Derogenes abba* n. sp. ex *Hippoglossoides platessoides*. A, whole body (Type-9562). B, terminal genitalia (Type-9562). C, hologenophore, forebody (Type-9563).

*Synonyms: Derogenes varicus* DV2 of Krupenko *et al*. [30]; *D. varicus* of Olson *et al*. [50].

*Type-host*: *Hippoglossoides platessoides* (Pleuronectiformes: Pleuronectidae), American plaice.

*Type-locality*: Svalbard, Norway, Arctic Ocean, at 79 59.860558 N, 15 27.879328 E, and 170 m depth.

*Other hosts*: Invertebrate hosts, first intermediate hosts: *Amauropsis islandica* (Naticidae), Iceland moonsnail; *Euspira pallida* (Naticidae), Pale moonsnail; *Buccinum scalariforme* (Buccinidae), ladder whelk (definitive host).

*Other localities*: United Kingdom, North Sea [50]. Keret Archipelago, White Sea. Dalniye Zelentsy, Barents Sea [30].

*Site in host*: Stomach.

*Deposited examined material:* Holotype (SMNH- Type-9562), 11 paratypes (SMNH- Type-9563–9573), and 2 paratypes with molecular information (hologenophores) (SMNH- Type-9563–9564) deposited in the Type collections of the Swedish Museum of Natural History (SMNH), Stockholm, Sweden. One paratype (TSZY-519) deposited in the collections of The Arctic University Museum of Norway (UiT), Tromsø, Norway.

*Paratypes with molecular information*: anterior parts of specimens mounted on a slide, posterior part used for molecular analysis: slide SMNH- Type-9563; slide SMNH- Type-9564.

*Representative DNA sequences*: Partial 28S, two sequences (GenBank, PP314018–PP314019); ITS2, two sequences (GenBank, PP314020, PP314022); Partial *cox*1, two sequences (GenBank PP384389–PP384390).

*Additional material examined for comparison*: Whole mounts: (1) Eighteen specimens of *D. varicus s. s*. ex *Salmo salar*, Bremanger, Norway, Northeast Atlantic (SMNH 218683–218700). (2). Ten specimens of *D. varicus s. s*. ex *Gadus morhua* from Svalbard, Norway, Arctic (TSZY-520–529). (3). *Derogenes varicus s. l*. from the collection of T. Odhner in the Invertebrates collection of the Swedish Museum of Natural History (SMNH): one specimen ex *Limanda limanda* from Gullmarsfjorden, Kristineberg, Sweden, NEA (SMNH-114551); one specimen ex *Argentina sphyraena* from Trondheim, Norway, NEA (SMNH-114558); one specimen ex *Molva molva* Trondheim, Norway, NEA (SMNH-114559); one specimen ex *Brosme brosme* from Trondheim, Norway, NEA (SMNH-114560); three specimens ex *Hippoglossus hippoglossus* from Trondheim, Norway, NEA (SMNH-104577); five specimens ex *Platichthys flesus* from Gullmarsfjorden, Kristineberg, Sweden, NEA (SMNH-208360).

*Etymology:* Named after ABBA, the Swedish pop supergroup renowned for hits like “*Dancing Queen*”, “*Chiquitita*” and “*Money*, *Money*, *Money*” which served as a source of entertainment for the first author during the creation of the illustrations. The group’s name is an acronym of the first letters of their first names arranged as a palindrome. Invariable, treated as a noun in apposition.

*Description*: Measurements and comparisons in Tables 3–5. Body stocky (Figs. 1A and 1B), nearly sausage-shaped; anterior and posterior ends rounded. Pre-oral lobe short. Oral sucker rounded. Pre-pharynx absent. Pharynx muscular. Oesophagus short. Intestines bifurcating anterior to sinus-organ. Intestinal caeca extending posteriorly to vitelline masses and terminating blindly. Ventral sucker rounded.

**Table 3:** Measurements of *Derogenes abba* n. sp. from *Hippoglossoides platessoides* off Svalbard and *Derogenes* spp. first described from the Atlantic. *Diameter. ^1^Note that *D. lacustris* is a freshwater species.

Species	*Derogenes abba* n. sp.	*D. varicus sensu stricto*	*D. bohaiensis* Qiu & Liang in Shen & Qiu, 1995	*D. crassus* Manter, 1934	*D. infirmus* (Linton, 1940)	*D. lacustris* ^1^ Tsuchida, Flores, Viozzi, Rauque & Urabe, 2021
Host	*Hippoglossoides platessoides*	*Salmo salar*	*Sebastes fuscescens*, *Pennahia argentata*	*Foetorepus agassizii*	*Cyclopterus lumpus*	*Galaxias maculatus* ^1^
Locality	Svalbard, Arctic Ocean	Bremanger, Norway, NEA	Yellow Sea and the Bo Hai Sea, China, NWA	Florida, USA, NWA	Massachusetts, USA, NWA	Lake Gutiérrez, Argentina
Source	Present study	Present study	[61]	[42]	[36]	[67]
Number	14	16	–	1	3	13
Body length	1100–1450 (1150)	1812–2661 (2276)	782–1003	2268	2340–2520	495–859
Body width	370–450 (450)	392–669 (528)	255–323	882	700–660	131–273
Forebody	396–485 (483)	739–937 (838) × 334–418 (385)	–	–	940–1170	–
Hindbody	350–400 (390)	698–930 (803) × 343–472 (382)	187–327	–	–	–
Preoral lobe length	20–38 (25)	57 (44–68)	–	–	–	–
Ventral sucker	226–425 (350) × 210–430 (355)	231–399 (353) × 268–434 (374)	238–372*	697*	490–420 × 490 – 350	113–200 × 103–203
Oral sucker	95–180 (150) × 110–225 (160)	223–335 (255) × 219–321 (247)	153*	285*	290–240 × 290–310	55–95 × 60–108
Pharynx	61–97 (75) × 64–93 (75)	79–112 (90) × 85–135 (101)	51*	–	70 × 110–100	28–50 × 35–60
Sinus sac	93–102 (95) × 84–99 (80)	93–135 (120) × 106–120 (113)	51–68 × 17–34	–	–	–
Seminal vesicle	56–80 (75) × 38–65 (54)	46–80 (70) × 30–75 (45)	68 × 51	–	–	–
Left testis	150–119 (154) × 84–110 (92)	152–208 (180) × 128–165 (145)	51 × 68	–	–	58–115 × 30–113
Right testis	136–148 (150) × 113–154 (78)	124–208 (164) × 120–180 (147)		–	–	–
Ovary	120–182 (143) × 110–168 (132)	128–180 (151) × 112–188 (155)	51*	–	–	45–88 × 43–80
Left vitelline mass	201–256 (228) × 136–207 (176)	201–256 (228) × 136–207 (176)	85 – 102 × 68	–	–	53–105 × 43–80
Right vitelline mass	181–280 (216) × 133–185 (162)	181–280 (216) × 133–185 (162)		–	–	–
Eggs	40–52 (48) × 28–34 (30)	40–53 (50) × 28–35 (31)	51–57 × 27–23	64 × 36	48–54 × 33	45–50 × 23–28

Abbreviations: NEA, Northeast Atlantic. NWA, Northwest Atlantic.

Male terminal genitalia in forebody. Sinus-sac oval to pyriform (Figs. 1B and 1C). Cone-shaped permanent muscular sinus-organ projecting into genital atrium. Ejaculatory duct and metraterm projecting into sinus-organ. Hermaphroditic duct thin-walled. Pars prostatica relatively short, lined by gland cells, leading to seminal vesicle. Seminal vesicle voluminous, oval, and thin-walled, situated in mid-forebody at considerable distance from ventral sucker. Testes oval to globular, symmetrical, posterior to ventral sucker.

Ovary globular, voluminous, post-testicular, sometimes overlapped by right vitelline mass. Laurer’s canal not observed. Vitelline masses in hindbody, round to oval, paired, situated on each side of body. Vitelline ducts fuse antero-medial to ovary. Seminal receptacle not observed. Uterus convoluted, uterine coils extending from near posterior extremity to sinus sac. Eggs oval. Excretory vesicle Y-shaped; branches reuniting dorsal to pharynx.

The morphology of the cercaria was described by Krupenko *et al*. [30].

#### *Derogenes* sp. (unpublished *Derogenes limula* of Looss, see discussion) (Figs. 2–4)

*Host*: *Parablennius tentacularis* (Blenniiformes: Blenniidae), tentacled blenny.

**Figure 2:**
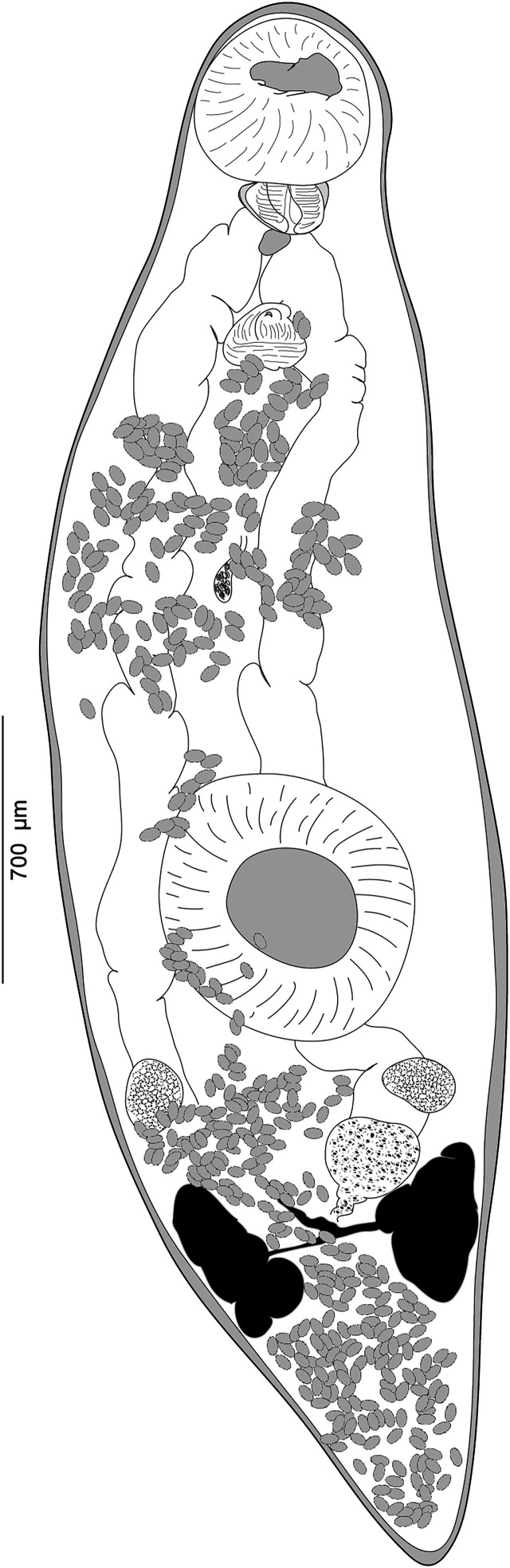
“*Derogenes limula*” ex *Parablennius tentacularis* (SMNH-208361).

*Locality*: Trieste, Italy, Central Mediterranean [25].

*Site in host*: Intestine.

*Material examined*: One specimen from *Parablennius tentacularis* off Trieste, Italy (SMNH-208361), from the collection of A. Looss in the Invertebrates collection of the Swedish Museum of Natural History (SMNH), Stockholm, Sweden (identified and labeled by A. Looss as *Derogenes* sp.). One specimen from *P. tentacularis* off Trieste, Italy (SMNH-222309), stained from the wet collection of A. Looss (894) at the SMNH; identified and labeled by A. Looss as “*Derogenes limula*”.

**Figure 3:**
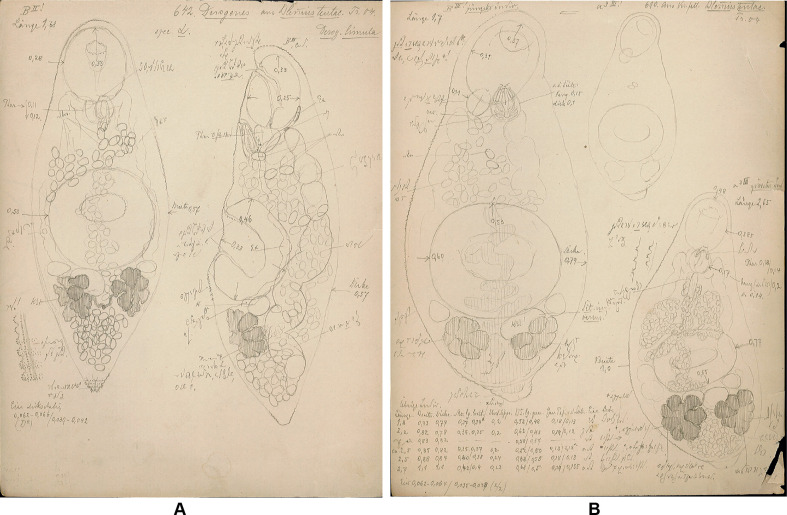
“*Derogenes limula*” ex *Parablennius tentacularis*. Unpublished line drawing by A. Looss.

**Figure 4:**
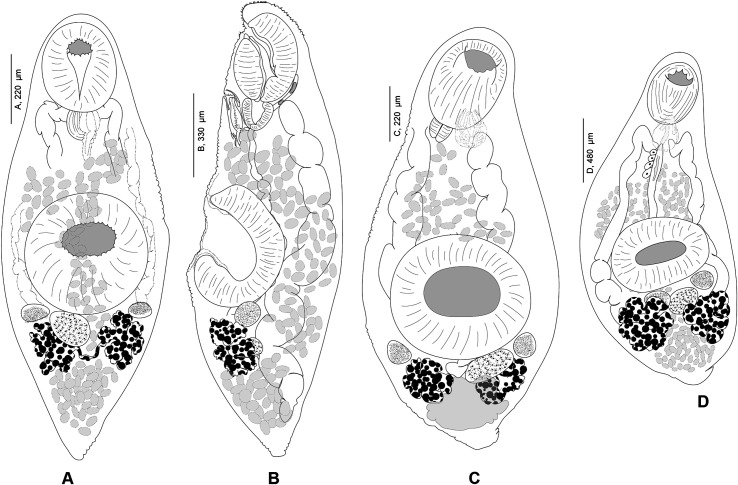
“*Derogenes limula*” ex *Parablennius tentacularis* based on A. Looss’s unpublished line drawings. A, Whole body, ventral view. B, Whole body, lateral view. C, Whole body, ventral view. D, Whole body, ventral view.

Archival documents: in addition to a single slide mounted by A. Looss (SMNH-208361) (Fig. 2), the archives include two unpublished line drawings (Figs. 3A and 3B), combined and reproduced in Figures 4A–4E.

*Description*: Measurements in Table 6. Body stocky, rounded anteriorly, pointed posteriorly (Figs. 4A, 4C, 4D), visibly larger at level of ventral sucker (Figs. 4C–4D). Tegument crenulate, with fine spines (Fig. 4B).

Oral sucker elongate, oval. Pharynx muscular, subglobular (Figs. 4A, 4C, 4D). Oesophagus short, caecal bifurcation at level of pharynx. Drüsenmagen absent (Fig. 4D). Caeca broad, extending to posterior region of hindbody, as far as uterus (Fig. 4B). Ventral sucker round to transversely oval, voluminous (Figs. 4A, 4C, 4D), larger than oral sucker.

Testes paired, oval, small, in hindbody, immediately posterior to ventral sucker (Fig. 4A). Ovary oval, voluminous, located between tested to slightly posteriorly to testes (Figs. 4A, 4C, 4D).

Vitellarium paired, in hindbody, posterior to testes; vitelline masses rosette-shaped, deeply lobed, connected one to another by a visible isthmus; composed both of 6–7 lobes (Figs. 4A, 4C, 4D). Uterine coils extending from posterior third of forebody to posterior end (Figs. 4A–4D), existing also in inter-vitelline masses space. Eggs numerous, large (Fig. 4A–4D). Excretory vesicle and position of bifurcation of stem not examined; excretory arms extending into forebody and unite dorsally to genital terminalia and dorsally to oral sucker.

*Remarks: Derogenes* sp. that we described above was found in A. Looss’s collection, labelled as “*Derogenes limula*”. As there are no published records of a species under that name, A. Looss probably intended to describe this *Derogenes* specimen from *P. tentacularis* as a new species, with the name “*D. limula*”. The eggs of this *Derogenes* sp. that we described above are over 40 μm and the species is thus consistent with “the large eggs group”. We compared the single specimen of *Derogenes* sp. (or “*D. limula*” as initially labeled by A. Looss) ex *P. tentacularis* to the Mediterranean congeneric *Derogenes* species. The present specimen *Derogenes* sp. ex *P. tentacularis* differs from *D. minor*, *D. robustus*, *D. affine*, and *D. latus* by its larger eggs. It resembles *D. ruber* in egg size (61 × 39 in *Derogenes* sp. *vs*. 62 × 39 in *D. ruber*) and in having lobed, tear-shaped vitelline masses. However, *Derogenes* sp. ex *P. tentacularis* can be readily distinguished from *D. ruber* by being smaller in all body measurements including the body (855 × 253 *vs*. 7869 × 1847). *Derogenes* sp. ex *P. tentacularis* can be easily distinguished from *D. varicus s. s*. and *D. abba* n. sp. by having lobed vitelline masses.

#### *Progonus muelleri* (Levinsen, 1881) (Figs. 5A and 5B)

*Type-host*: *Myoxocephalus scorpius* (as *Cottus scorpius*) (Perciformes: Cottidae), shorthorn sculpin [35].

**Figure 5:**
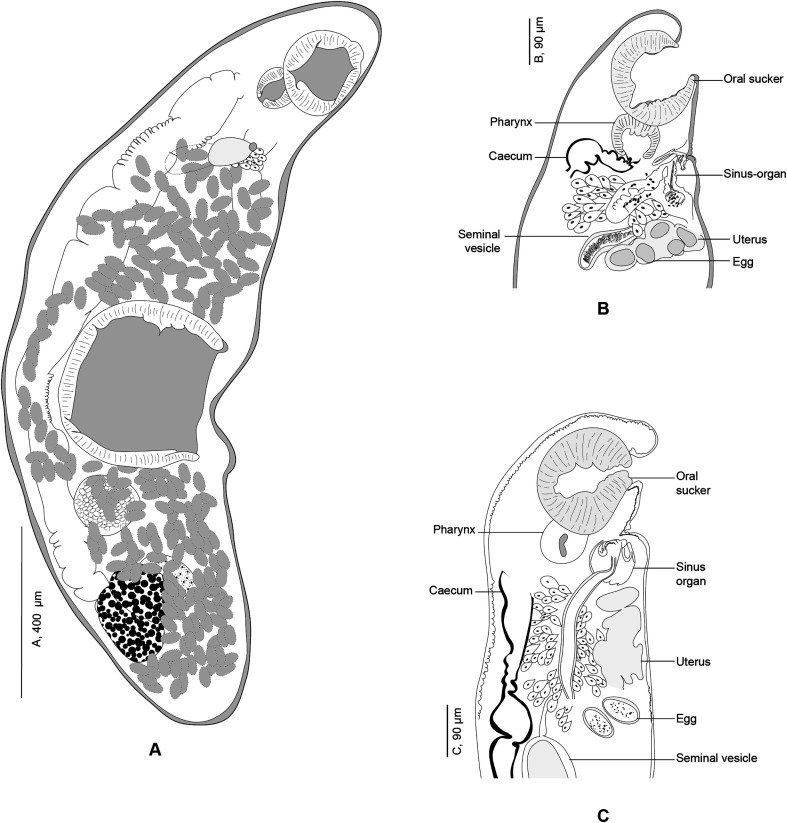
*Progonus muelleri* (Levinsen, 1881) ex *Myoxocephalus scorpius* (NHMD-114950) and comparison between the terminal genitalia in *Progonus* and in *Derogenes*. A, *P. muelleri* ex *M. scorpius* (NHMD-114950), whole body. B, *P. muelleri* ex *M. scorpius*, terminal genitalia, horizontal section (SMNH-114586). C, *Derogenes varicus sensu stricto* ex *Limanda limanda*, terminal genitalia, horizontal section (SMNH-114557).

*Other hosts*: *Gadus macrocephalus* (as *Gadus ovak*) (Gadiformes: Gadidae), Greenland cod [35].

*Type-locality*: Aasiaat [Egedesminde], Greenland [35].

*Site in host*: Stomach.

*Deposited examined material*:

*Whole mounts*: Two specimens of *P. muelleri* ex *Myoxocephalus scorpius* from Aasiaat [Egedesminde], Greenland, deposited at the Natural History Museum of Denmark (SNM), Copenhagen, Denmark (NHMD-114950/ old catalogue number ZMUC-TRE-000032), Paralectotypes.

*Transverse sections*: T. Odhner’s collections in the Invertebrates collection of the Swedish Museum of Natural History (SMNH), Stockholm, Sweden; two specimens ex *Myoxocephalus scorpius* from Spitsbergen, Svalbard, Norway, Arctic (SMNH 114584–114585) (identified and labeled by T. Odhner as *P. muelleri*); One specimen from the same host and locality (SMNH 114587) (identified and labeled by T. Odhner as *Genarches muelleri* (Levinsen, 1881)).

*Longitudinal sections*: T. Odhner’s collections in the Invertebrates collection of the SMNH; one specimen ex *Myoxocephalus scorpius* from Spitsbergen, Svalbard, Norway, Arctic (SMNH 114586) (identified and labeled by T. Odhner as *P. muelleri*).

*Additional material examined for comparison*: *Transverse sections*: T. Odhner’s collections in the Invertebrates collection of the SMNH: four specimens of *D. varicus s. s*. ex *Limanda limanda* from Gullmarsfjorden, Kristineberg, Sweden, NEA (SMNH 114554–114557) (identified and labeled by T. Odhner as *D. varicus*).

*Redescription:* Body elongated, fusiform, rounded anteriorly (Fig. 5A). Tegument smooth, unarmed. Pre-oral lobe present. Oral sucker subterminal, small, rounded. Prepharynx not observed. Pharynx subglobular. Oesophagus not observed. Intestinal bifurcation in anterior third of forebody. Caeca wide, extending to and joining in anterior hindbody, forming cyclocoel, approximatively at level of testis. Ventral sucker subglobular, voluminous, clearly larger than oral sucker.

Genital pore a rounded pit, posterior to pharynx. Sinus sac oval to fusiform. Sinus-organ muscular, conical (Fig. 5B). Hermaphroditic duct thin walled. Seminal vesicle located in mid-forebody, saccular, fusiform. Pars prostatica short, lined with glandular cells. Testes two, oval, immediately posterior to ventral sucker.

Ovary globular, immediately post testicular. Uterus dorsal, extending to genital terminalia. Metraterm in midforebody, muscular. Eggs oval. Vitelline masses two, postovarian, sub-globular. Excretory pore posterior, terminal. Excretory vesicle not detected. Excretory branches joining in forebody, dorsally to pharynx.

*Remarks*: The type-material received from Natural History Museum of Denmark (SNM), Copenhagen, Denmark includes nine specimens preserved in 70% ethanol from *M. scorpius* off Aasiaat [Egedesminde], Greenland, and were indicated as the Holotype. Two specimens were stained in acetocarmine, mounted on slides, and designated herein as Paralectotypes. Comparative terminal genitalia of *D. varicus s. s*. shown in Figure 5C.

### Molecular characterisation

**Figure 6:**
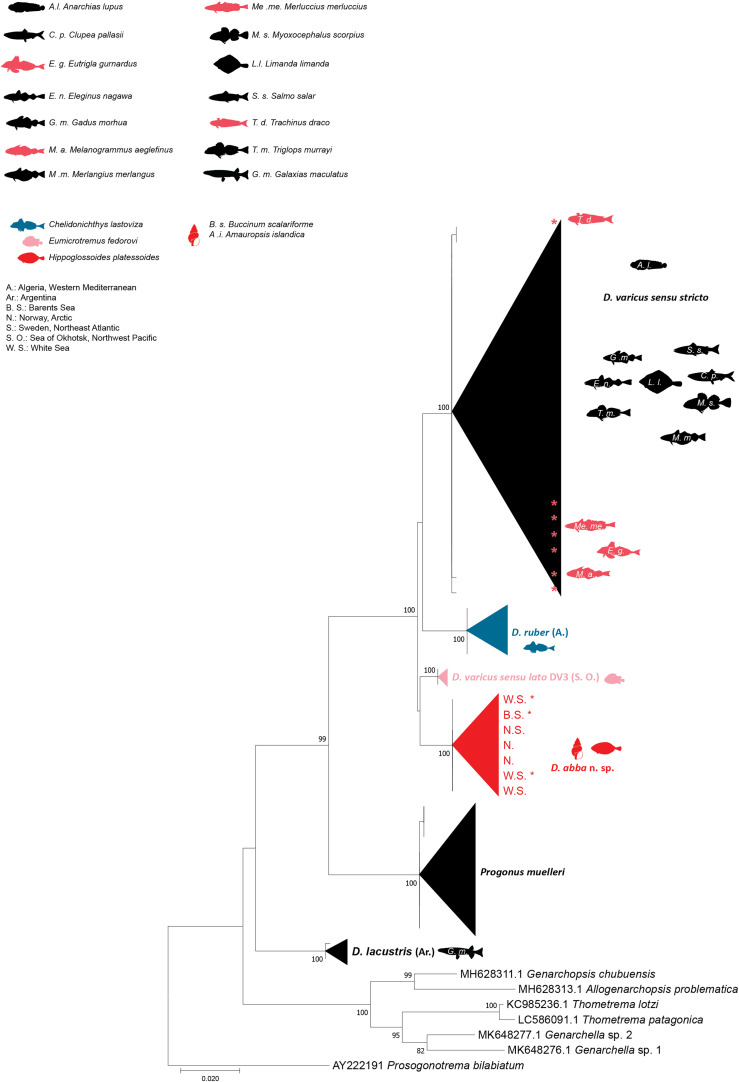
Tree inferred using the maximum likelihood method based on the 28S rDNA sequence data; only bootstrap values higher than 70 are indicated. The newly generated sequences of *Derogenes varicus sensu stricto* from Sweden, Northeast Atlantic are indicated by*. Other *D. varicus sensu stricto* are those of Krupenko *et al*. [30] from the Barents Sea and White Sea; and those of Bouguerche *et al*. [3] from Northeast Atlantic, off Sweden and Norway and from Svalbard, Norway, Arctic Ocean.

**Figure 7:**
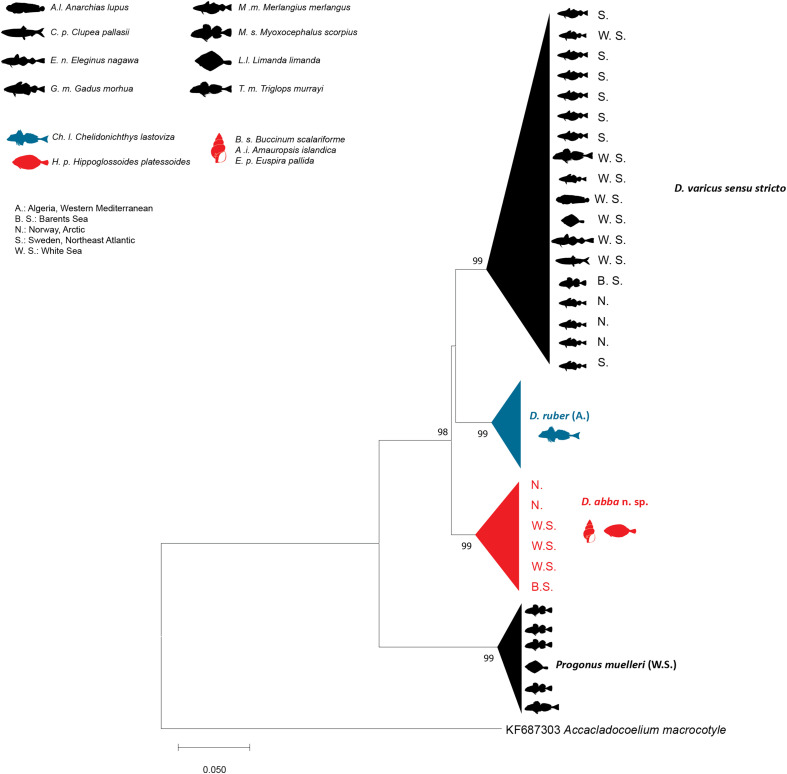
Tree inferred using the maximum likelihood method based on the ITS2 sequence data; only bootstrap values higher than 70 are indicated.

**Figure 8:**
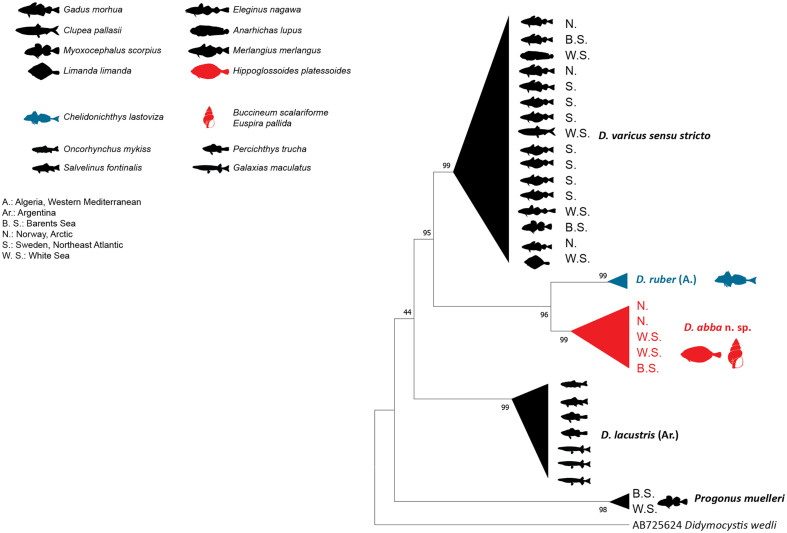
Tree inferred using the maximum likelihood method based on the *cox*1 sequences; only bootstrap values higher than 70 are indicated.

The NJ and ML methods led to similar tree topologies and thus only the ML trees are shown (Figures 6–8).

The 28S dataset included 53 nucleotide sequences of the Derogenidae Nicoll, 1910. The trimmed matrix comprised 642 positions. The newly generated sequences of *D. varicus* ex *M. aeglefinus*, *E. gurnardus*, *T. draco*, and *M. merluccius* from Sweden were identical and clustered within the *D. varicus s. s*. clade without any host-related structuring.

The two newly generated 28S sequences for *D. abba* n. sp. ex *H. platessoides* were identical to each other and to sequences of rediae of *D. varicus* DV2 of Krupenko *et al*. [30] ex the gastropod intermediate mollusc hosts *Euspira pallida* and *Amauropsis islandica* from the White Sea and the Barents Sea; they were also identical to adults ex *B. scalariforme* from the White Sea and from the definitive host *H. platessoides* from the North Sea. All sequences divergence of *D. abba* n. sp. ex *H. platessoides* from *D. varicus s. s*. (= DV1 [26]) from these hosts from the White Sea, Barents Sea (Russia) and Scandinavian waters of the North Sea (Sweden and Norway) was 3%. There was 2% divergence between the sequences of *D. abba* n. sp. and those of *D. varicus* DV3 [30] recovered from *E. fedorovi* in the North Pacific [63]. They differed from those of *D. ruber* ex *Chelidonichthys lastoviza* off the coast of Algeria by 3%. The largest divergence was shown between the sequences of *D. abba* n. sp. and those of *D. lacustris* from Argentinean freshwater fishes [67], reaching up to 10%. The divergence between *D. abba* n. sp. and *P. muelleri* from different White Sea fishes [30] was 8%.

In the phylogenetic tree of 28S rDNA sequences (Fig. 6), *D. abba* n. sp. from Svalbard (ex *H. platessoides*) clustered as a well-supported clade with *D. varicus* DV2 ex the same host from the North Sea [46] and ex *B. scalariforme* from the White Sea [30]. This clade was well separated from the *D. varicus s. s*. clade ex multiple hosts from the White Sea, Barents Sea (Russia) and Scandinavian waters of the North Sea (Sweden and Norway), the DV3 clade (ex *E. fedorovi*), the *D. lacustris* clade (ex *G. maculatus*), and the *D. ruber* clade (ex *C. lastoviza*).

The ITS2 tree was constructed using 36 sequences of the Derogenidae (Fig. 7). The trimmed matrix included 428 positions. The newly generated sequences of *D. abba* n. sp. from Svalbard (ex *H. platessoides*) were identical to those of *D. varicus* DV2 ex intermediate hosts from the White Sea and Barents Sea (Russia). They differed by 4% from the sequences of *D. varicus s. s*. from the White Sea, Barents Sea (Russia), and Scandinavian waters of the North Sea (Sweden and Norway) and those of *D. ruber* off Algeria (ex *C. lastoviza*). The sequences of *D. abba* n. sp. from Svalbard (ex *H. platessoides*) formed a well-supported clade with those of *D. varicus* DV2. This clade separated from the *D. varicus s. s*. clade and the *D. ruber* clade.

The *cox*1 sequences of *D. abba* n. sp. from Svalbard (ex *H. platessoides*) were aligned with 31 other derogenid sequences, all relating to the genera *Derogenes* and *Progonus*. The trimmed matrix included 783 positions. Two of them were identical, while these sequences differed by 1% from those of *D. varicus* DV2 from the White and Barents seas off Russia (ex intermediate hosts). The divergence was 21–31% between the sequences of *D. abba* n. sp. and those of *D. varicus s. s*. [3, 30] and was 21–24% between the former and those of *D. ruber* from Algerian *C. lastoviza* [18]. The highest divergence to a congener was 38–39% from *D. lacustris* from Argentinean freshwater fishes [67]. In the phylogenetic tree of *cox*1 (Fig. 8), the sequences of *D. abba* n. sp. clustered with those of *D. varicus* DV2 within a well-supported clade. This clade was well separated from the *D. ruber* clade and the *D. lacustris* clade.

## Discussion

### Differential diagnosis for *Derogenes abba* n. sp.

WoRMS [71] lists 26 species of the genus *Derogenes* from the Mediterranean, Atlantic, Pacific, Indian Ocean and the Antarctic. *Derogenes bohaiensis* Qiu & Liang, 1995, *D. crassus* Manter, 1934, *D. infirmus* (Linton, 1940), *D. lacustris* Tsuchida, Flores, Viozzi, Rauque & Urabe, 2021, and *D. varicus* (Müller, 1784) were all first described from Atlantic waters [36, 42, 46, 59, 61, 67]. *Derogenes adriaticus* Nikolaeva, 1966, *D. affinis* (Rudolphi, 1819), *D. bonnieri* (Monticelli, 1893), *D. latus* Janiszewska, 1953, *D. minor* Looss, 1901, *D. ruber* Lühe, 1900, and *D. robustus* Brinkmann, 1966 were all first described from the Mediterranean [9, 25, 39, 40, 44, 48, 55]. *Derogenes capricorniensis* Bray, 1989, *D. chelidonichthydis* Shen, 1989, *D. epinepheli* Wang, 1982, *D. magnus* Wang, 1991, *D. gadi* Shen, 1990, *D. macrostoma* Yamaguti, 1938, *D. nototheniae* Manter, 1954, *D. pharyngicola* Bray, Cribb & Barker, 1993, *D. minoi* Shen, 1990, and *D. pearsoni* Bray, Cribb & Barker, 1993 were all first described from the Pacific [5, 7, 43, 58, 60, 69, 70, 72]. *Derogenes hyderabadensis* Jaiswal, 1967 and *D. indicus* (Singh, 1979) were described from the Indian Ocean [24, 62]. The only known species from the Antarctic is *D. johnstoni* Prudhoe & Bray, 1973 [54]. Their hosts, collection localities, number of specimens measured and morphometrical data are summarised in Tables 3–5. According to Bray *et al*. [7], the species of *Derogenes* can be allocated to two groups, based on egg size. We updated the lists given by Tsuchida *et al*. [67], and the 26 *Derogenes* species accepted on WoRMS [71] can be divided as follows: the small egg group with eggs <40 μm comprises eight species: *D. adriaticus*, *D. capricorniensis*, *D. indicus*, *D. epinepheli*, *D. macrouri*, *D. nototheniae*, *D. pearsoni*, and *D. pharyngicola*. The large egg group comprises 17 species with eggs >40 μm which are: *D. affine*, *D. bohaiensi*s, *D. chelidonichthydis*, *D. crassus*, *D. fuhrmanni*, *D. gadi*, *D. johnstoni*, *D. latus*, *D. infirmus*, *D. macrostoma*, *D. magnus*, *D. minoi*, *D. minor*, *D. ruber*, *D. robustus*, *D. parvus*, and *D. varicus*. For *D. bonnieri* only body length was given [44], and thus we cannot assign it to any group. As eggs of *D. abba* n. sp. are over 40 μm, it is here considered relative to the second group. Krupenko *et al*. [30] provided morphometric data from molecularly identified adult *D. varicus s. s*. and a single *D. varicus* DV2. *Derogenes abba* n. sp. ex *H. platessoides* from Svalbard were similar to the specimen studied by Krupenko *et al*. [30], but egg dimensions differed. The eggs of the single *D. varicus* DV2 specimen from the stomach of the whelk *B. scalariforme* measured 57–63 × 32–38 μm, while those from the present specimens were smaller (40–52 × 28–34 μm). However, the trematodes from the White- and Barents Sea gastropods and those from Svalbard *H. platessoides* showed no difference in 28S or ITS2 sequences and differed only by a single substitution in the *cox*1 gene. *Derogenes abba* n. sp. ex *H. platessoides* differed from *D. varicus s. s*. by 3% in 28S, by 4% in ITS2, and by 21–31% in *cox*1. This is strong evidence that *D. abba* n. sp. ex *H. platessoides* and *D. varicus s. s*. are distinct species.

In the following section, we compare the new species *D. abba* n. sp. with five species of the large egg group first described from the Atlantic (Table 3), five species first described from the Mediterranean (Table 4), and seven species first described from the Pacific, Antarctic, and Indian Oceans (Table 5).

**Table 4:** Measurements of *Derogenes abba* n. sp. from *Hippoglossoides platessoides* off Svalbard and *Derogenes* spp. first described from the Mediterranean. *Diameter. ^1^We included measurements from the redescription of Rudolphi’s specimen by Lühe (1901) as the original description did not provide any measurements. ^2^Ratio given by Brinkmann (1967). For the 6th Mediterranean species *D. bonnieri* (Monticelli, 1893) from *Eutrigla gurnardus* off Wimereux, France [44], only body length (1500–2000) was given.

Species	*Derogenes abba* n. sp.	*D. affine* (Rudolphi, 1819)	*D. latus* Janiszewska, 1953	*D. minor* Looss, 1901	*D. robustus* Brinkmann, 1967	*D. ruber*
Host	*Hippoglossoides platessoides*	*Scorpaenopsis cirrosa* ^1^	*Mullus barbatus*	*Labrus merula*	*Serranus scriba*	*C. lastoviza*
Locality	Svalbard, Arctic Ocean	Rimini, Italy, WM	Split, Croatia, CM	Trieste, Italy, WM	Rhodes, Greece, CM	Trieste, Italy, WM
Source	Present study	[41]^1^	[25]	[3]	[9]	[3]
Number of specimens	14	1	–	1	1	2
Body length	1100–1450 (1150)	1400	–	2022	2600	7869
Body width	370–450 (450)	400	–	477	850	1847
Forebody	396–485 (483)	–	–	983 × 500	–	3765 × 1714
Hindbody	350–400 (390)	–	–	706 × 366	–	2750 × 959
Preoral lobe length	20–38 (25)	–	–	22	–	88
Ventral sucker	226–425 (350) × 210–430 (355)	250*	800 × 630	349 × 321	550 *	1257 × 123
Oral sucker	95–180 (150) × 110–225 (160)	190*	500 × 450	222 × 207	250*	655 × 708
Sucker-L R		100*	–	–	2 : 3*^,2^	
Sucker-W R		–	–	–	–	–
Pharynx	61–97 (75) × 64–93 (75)	–	180 × 160	91 × 96	–	254 × 275
Sinus sac	93–102 (95) × 84–99 (80)	–		97 × 92	–	289 × 291
Seminal vesicle	56–80 (75) × 38–65 (54)	–	310 × 90	119 × 32	–	291 × 134
Left testis	150–119 (154) × 84–110 (92)	–	340 × 190	115 × 88	–	271 × 353
Right testis	136–148 (150) × 113–154 (78)	–		136 × 101	–	−256 × 280
Ovary	120–182 (143) × 110–168 (132)	–	340 × 110	129 × 107	–	–
Left vitelline mass	201–256 (228) × 136–207 (176)	–	540 × 350	180 × 103	–	546 × 658
Right vitelline mass	181–280 (216) × 133–185 (162)	–		204 × 109	–	550 × 446
Eggs	40–52 (48) × 28–34 (30)	45 × 20	56 × 26	58 × 39	55 × 30	62 × 39

Abbreviations: CM, Central Mediterranean. WM, Western Mediterranean.

**Table 5:** Measurements of *Derogenes abba* n. sp. from *Hippoglossoides platessoides* off Svalbard and *Derogenes* spp. first described from the Pacific, Antarctic, and Indian Ocean. *Diameter.

Species	*Derogenes abba* n. sp.	*D. chelidonichthydis* Shen, 1989	*D. gadi* Shen, 1990	*D. macrostoma* Yamaguti, 1938	*Derogenes magnus* Wang, 1991	*D. minoi* Shen, 1990	*D. johnstoni* Prudhoe & Bray, 1973	*D. hyderabadensis* Jaiswal, 1967
Host	*Hi. Platessoides*	*Chelidonichthys kumu*	*Gadus macrocephalus*	*Inimicus japonicus*	*Mugil cephalus*	*Minous monodactylus*	*Artedidraco shackletoni*	*Channa punctata*
Locality	Svalbard, Arctic Ocean	Jiaozhou Bay, China, NWP	Yellow Sea, China, NWP	Fukui, Japan, NWP	Fujian, China, NWP	Changjiang Estuary, China, NWP	Antarctic region	Hyderabad, India, Indian Ocean
Source	Present study	[60]	[58]	[72]	[69]	[59]	[54]	[24]
N.	14	–	1	2	–	1	2	1
Body length	1100–1450	2091	3332	3000–3200	4000	2346	1700–2400	2560
Body width	370–450	527	748	700–950	1200	748	600–840	650
Forebody	396–485	–	–	–	–	1088	–	–
Hindbody	350–400	–	–	–	–	663	–	–
Preoral lobe length	20–38	51	–	24–80	–	34	25–28	–
Ventral sucker	226–425 × 210–430	374	561*	750–800*	640 × 618	561 × 510	500–530	570 × 530
Oral sucker	95–180 × 110–225	153 × 187	221 × 255	440–460 × 510	336 × 352	340 × 408	^1^	320 × 280
Pharynx	61–97 × 64–93	85 × 102	119	150 × 210	160 × 152	136 × 170	140 × 100	–
Sinus sac	93–102 × 84–99	85 × 65	170 × 136	200*		221 × 102	–	–
Seminal vesicle	56–80 × 38–65	119 × 85	–	–	192 × 128		–	–
Left testis	150–119 × 84–110	68 × 51	221 × 136	160–200 × 250–275	240 × 224	119 × 204	–	160 × 150
Right testis	136–148 × 113–154	–			288 × 240	88 × 153	–	210 × 110
Ovary	120–182 × 110–168	119	170 × 204	188–225 × 250–260	480 × 192	170*	–	210 × 130
Left vitelline mass	201–256 × 136–207	170 × 119	306 × 289	250–310 × 150–240	400 × 240	289 × 136	–	180 × 70
Right vitelline mass	181–280 × 133–185	–	–	–	352 × 240		–	180 × 130
Eggs	40–52 × 28–34	48–57 × 30–33	48–51 × 27–30	57–66 × 36–42	60–66 × 35–36	51–60 × 30–36	–	31–41 × 14–16

Abbreviations: NWP, Northwest Pacific; N., number of specimens.

#### Comparison with Atlantic species (Table 3)

*Derogenes abba* n. sp. can be distinguished from *D. bohaiensis* by having a larger body (1100–1450 × 370–450 *vs*. 782–1003 ×255–323), and a larger sinus sac (93–102 × 84–99 *vs*. 51–68 × 17–34). It can also be distinguished from *D. bohaiensis* by having Drüsenmagen which are lacking in *D. bohaiensis*. *Derogenes abba* n. sp. differs from *D. crassus* by having a smaller body (1100–1450 × 370–450 *vs*. 2268 × 882), smaller ventral (350 *vs*. 697) and oral (150 *vs*. 285) suckers, and by the extension of uterine coils which fills most of the body in *D. crassus vs*. not extending to body edges in *D. abba* n. sp. *Derogenes crassus* can be easily differentiated from *D. abba* n. sp. by its vitellaria extended transversally rather than longitudinally. *Derogenes abba* n. sp. differs from *D. infirmus* by forebody length (396–485 *vs*. 940–1170), and in having slightly smaller oral (95–180 × 110–225 *vs*. 290–240 × 290–310) and ventral (226–425 × 210–430 *vs*. 420–490× 359–490) suckers. They can be easily distinguished by the lack of post-gonadal uterine distribution in *D. infirmus*.

*Derogenes abba* n. sp. can be distinguished from *D. lacustris* by having a longer pars prostatica, larger oral sucker, larger pharynx, larger vitelline masses, and visibly larger eggs (see Tables 4 and 5 in Tsuchida *et al*. [67]). In addition, the divergence between *D. abba* n. sp. and *D. lacustris* ranged between 10% in 28S and reached 38–39% in *cox*1, in addition to reciprocal monophyly in both trees (Figs. 6 and 8).

As discussed below, we could find no morphological basis for the separation of *D. abba* n. sp. from *D. varicus s.s*.

#### Comparison with Mediterranean species (Table 4)

*Derogenes abba* n. sp. differs from *D. affinis* by having larger oral (95–180 × 110–225 *vs*. 190) and ventral (226–425 × 210–430 *vs*. 250) suckers and larger eggs (40–52 × 28–34 *vs*. 45 × 20). *Derogenes abba* n. sp. differs from *D. latus* by having markedly smaller body organs (see Table 4). These species also differ in the vitellaria shape, which are deeply lobed in *D. latus vs*. entire in *D. abba* n. sp.; and by the posterior end, being rounded in our specimens *vs*. sharply narrowing in *D. latus*. *Derogenes abba* n. sp. most closely resembles *D. minor* in the position of the seminal vesicle, length of pars prostatica, and the position and dimensions of the suckers but the two are distinguished by the posterior end being pointed in *D. minor vs*. rounded in *D. abba* n. sp and by the shape of vitelline masses, being lobed and “mulberry shaped” in *D. minor vs*. entire in *D. abba* n. sp. *Derogenes abba* n. sp. shares a sausage-shaped body with *D. robustus*. *Derogenes abba* n. sp differs from *D. robustus* by being smaller (1100–1450 × 370–450 *vs*. 2600 × 850) with a smaller ventral sucker (226–425 × 210–430 *vs*. 550), the anterior extent of uterine coils (present in mid-forebody in *D. abba* n. sp. *vs*. absent in mid-forebody in *D. robustus*) and the shape of seminal vesicle (saccular in *D. abba* n. sp. *vs*. sausage-shaped in *D. robustus*).

*Derogenes abba* n. sp. can be distinguished from *D. ruber* by the position of the testes (preovarian in *D. ruber vs*. postovarian in *D. abba* n. sp.) and by the lobed vitellarium of *D. ruber* being lacking in *D. abba* n. sp. *Derogenes abba* n. sp. and *D. ruber* can also be distinguished by the body length (1100–1450 in *D. abba* n. sp. *vs*. 5000–6000 in *D. ruber*), by having much smaller suckers (oral, 95–180 in *D. abba* n. sp. *vs*. 600 in *D. ruber*; ventral, 226–425 in *D. abba* n. sp. *vs*. 750 in *D. ruber*) and smaller vitelline masses in *D. abba* n. sp. The genetic divergence between *D. abba* n. sp. and *D. ruber* ranged from 3% in 28S and to 21–24% in *cox*1. The two species also clustered in distinct clades (Figs. 6–8).

#### Comparison with species from the Pacific (Table 5)

*Derogenes abba* n. sp. differs from *D. minoi* in smaller body size and organs (see Table 5). The two species differ by the indented vitelline masses in *D. minoi vs*. unlobed masses in *D. abba* n. sp. *Derogenes magnus* resembles *D. abba* n. sp. in also having a sausage-shaped body. *Derogenes abba* n. sp. can be distinguished from *D. magnus* by being smaller (1100–1450 × 370–450 *vs*. 4000 × 1200), with smaller suckers, pharynx, seminal vesicle testes, ovary, and vitelline masses.

*Derogenes abba* n. sp. differs from *D. chelidonichthydis* by having a smaller body (1150 *vs*. 2091), smaller pharynx (75 × 75 *vs*. 50 × 102), larger sinus-sac (95 × 80 *vs*. 85 × 65), smaller seminal vesicle (75 × 54 *vs*. 119 × 85), and larger testes. The two species can also be distinguished by the posterior end being sharply pointed in *D. chelidonichthydis* vs rounded in *D. abba* n. sp.

From *D. gadi*, *D. abba* n. sp. can be differentiated by having a shorter body (1100–1450 *vs*. 3332), a shorter forebody, a shorter pars prostatica, a smaller ventral sucker (226–425 *vs*. 561), a smaller ovary (120–182 × 110–168 *vs*. 170 × 204) and smaller vitelline masses.

*Derogenes abba* n. sp. differs from *D. macrostoma* by having a smaller body (1100–1450 × 370–450 *vs*. 3000–3200 × 700–950), smaller oral (95–180 × 110–225 *vs*. 440–460 × 510) and ventral (226–425× 210–430 *vs*. 750–800) suckers, and a smaller ovary (120–182 × 110–168 *vs*. 188–225 × 250–260).

#### Comparison with species from the Antarctic (Table 5)

*Derogenes abba* n. sp. differs from *D. johnstoni* by having a smaller ventral sucker (226–425× 210–430 *vs*. 500–530), smaller eggs (length 40–63 *vs*. 62–72) and by the shape and size of the seminal vesicle (oval, 56–80 × 38–65 *vs*. elongate, 360 × 120).

#### Comparison with species from the Indian Ocean (Table 5)

*Derogenes abba* n. sp. differs from *D. hyderabadensis* by having a smaller body (1147–1882 × 216–499 *vs*. 2560 × 650), smaller oral (130–223 × 132–235 *vs*. 320 × 280) and ventral (251–384 × 251–359 *vs*. 570 × 530) suckers, smaller vitelline masses (see Table 5) and larger eggs (48–58 × 30–52 *vs*. 31–41 × 14–16). It can also be distinguished by having the uterine coils reaching the post-vitelline field, whereas post-vitelline uterine coils are lacking in *D. hyderabadensis*.

#### *Derogenes abba* n. sp. as a cryptic species

Genetically, Krupenko *et al*. [30] split *D. varicus* species complex into 4 groups: DV1–DV4. Bouguerche *et al*. [3] strongly showed that the DV1 clade is the “real” *D. varicus* and proposed to recognise it as *D. varicus s. s*. through the analysis of the 28S sequences of *D. varicus* from the type-host, the Atlantic salmon *S. salar*. Krupenko *et al*. [30] did not find any adult stages of DV2 in fish and hence could not provide further morphological comparison of DV1 and DV2. Additionally, the only available sequences of *D. varicus s. s*. from the type-host had been made available only recently [3]. Herein, the sequences of *D. abba* n. sp. from Arctic *H. platessoides* were identical to those of *D. varicus s. l*. from the same host in the North Sea [50] and those of *D. varicus* DV2 as rediae from gastropods in the White and Barents seas [30]. They were also identical to *D. varicus* of Olson *et al*. [50] (AY222189) ex *H. platessoide*s from the North Sea. On the other hand, they differed from the sequence of *D. varicus s. s*. from Norwegian *S. salar* by 3% as *p*-distance. This level of divergence is comparable to that observed between the well-established Mediterranean species *D. ruber* ex *C. lastoviza* from the Western Mediterranean, off Algeria (3% as well). Thus, the genetic divergence supports that *D. abba* n. sp. is a separate species from *D. varicus s. s*. This species uses a different first intermediate host, and produces cercariae that can be morphologically distinguished from those of *D. varicus s. s*. [30].

Krupenko *et al*. [30] speculated that *D. varicus* DV2 is the species that Shulman and Shulman-Albova (1953) called *D. crassus* Manter, 1934. Type localities for *D. crassus sensu* Manter (1934) and of *D. abba* n. sp. are so distinct (Tortugas, Florida, western Atlantic for *D. crassus* [42] *vs*. Svalbard, Arctic Norway, for *D. abba* n. sp.) that we consider this unlikely. However, it remains possible that *D. crassus sensu* Shulman and Shulman-Albova (1953) represents *D. abba* n. sp.

The lineage *D. varicus* DV4 corresponds to an 18S rDNA sequence (AJ287511) of *D. varicus* of Littlewood and Olson [37] from the same host as *D. abba* n. sp., *H. platessoides* from the North Sea. DV4 was labeled only based on the genetic divergence of 0.13% (*p*-distance) from *D. abba* n. sp. (DV2) in the 18S rDNA sequence [30]. In light of the available data, it is premature to consider *D. varicus* DV4 as a distinct species from *D. abba* n. p. considering that our study does not include the 18S rDNA analysis. Hence, we cannot estimate the taxonomical position of DV4 further. Meanwhile, the possibility that *D. varicus* (*s.s.)* also occurs in *H. platessoides* is not ruled out.

Overall, the evolution of understanding of this genus is such that while most combinations of species can be distinguished on the basis of morphology, some are presently morphologically cryptic with respect to each other. On this basis, it remains critical that further study is based on both morphological and molecular data. A key area of uncertainty relates to patterns of host-specificity. Some species, e.g. *D. varicus s.s.*, appear to be genuinely euryxenic, whereas several others apparently have highly restricted host ranges. The extent to which this is a genuine reflection of the true nature of these species is uncertain. Certainly, for clearly closely related species, it is not obvious why host-specificity patterns should differ so dramatically.

### The “lost collections of A. Looss”: Mediterranean *Derogenes* spp.

The German parasitologist Prof. Arthur Looss (1861–1923) was among one of the most prolific parasitologists and taxonomists and was known for his “*enthusiasm and energy as a researcher that have probably seldom been surpassed*, *especially a painstaking attention to detail that is unfortunately rare*” [33]. After his death, his collection was divided between numerous institutions: Smithsonian National Museum of Natural History in Washington (USA), the Natural History Museum in Berlin and the Natural History Museum in Leipzig (Germany), Gothenburg Museum of Natural History and the Swedish Museum of Natural History (Sweden) (see Kuzmina and Holovachov [31]). A part of Looss’s collection including his archives was sold to the Swedish Museum of Natural History (Naturhistoriska riksmuseet) in Stockholm by his widow, Elise Looss in 1924 [31] and includes slides of which some are actually type material and vials containing trematodes preserved in ethanol, along with several publication-ready drawings and original line drawings.

One of the intriguing derogenids that we encountered in this collection, is “*D. limula*” which we described above as *Derogenes* sp., ex *P. tentacularis*, collected from off Trieste, Italy, Central Mediterranean. Curiously, we also found line drawings by A. Looss (see Figs. 3A and 3B) labeled as “*Derogenes limula*”, which suggests that he intended to describe *Derogenes* from *P. tentacularis* as a new species, with the name “*D. limula*”. We found one specimen (SMNH 208361) for which the measurements are presented in Table 6. The eggs of this “*D. limula*” that we described above as *Derogenes* sp. are over 40 μm and the species is thus consistent with the “large eggs group”. We compared the measurements of the sole specimen of *Derogenes* ex *P. tentacularis* to those of congeneric species from the Mediterranean (Table 6). The single specimen of *Derogenes* sp. (or “*D. limula*” as initially referred to by A. Looss on the illustrations) ex *P. tentacularis* differs from *D. minor*, *D. robustus*, *D. affine*, and *D. latus* by its larger eggs. It resembles *D. ruber* in egg size (61 × 39 in *Derogenes* sp. *vs*. 62 × 39 in *D. ruber*) and in having lobed, tear-shaped vitelline masses. However, *Derogenes* sp. ex *P. tentacularis* differ from *D. ruber* by being smaller in all body measurements including the body (855 × 253 *vs*. 7869 × 1847). We note though that body size is not a sufficient differentiating character and can vary with the age of the worm and the suitability of the host.

**Table 6:** Measurements of *Derogenes* sp. “*limula*”. from *Parablennius tentacularis* off Trieste, Italy and *Derogenes* spp. first described from the Mediterranean. *Diameter. ^1^We included measurements from the redescription of Rudolphi’s specimen by Lühe (1901) as the original description did not provide any measurements.

Species	*Derogenes* sp. “*limula*”	*D. affine* (Rudolphi, 1819)	*D. latus* Janiszewska, 1953	*D. minor* Looss, 1901	*D. ruber* Lühe, 1900	*D. robustus* Brinkmann, 1967
Host	*Parablennius tentacularis*	*Scorpaenopsis cirrosa* ^1^	*Mullus barbatus*	*Labrus merula*	*C. lastoviza*	*Serranus scriba*
Locality	Trieste, Italy, CM	Rimini, Italy, CM	Split, Croatia, CM	Trieste, Italy, CM	Trieste, Italy, CM	Rhodes, Greece, CM
Source	Present study	[41]^1^	[25]	[3]	[3]	(Brinkmann, 1967)
Number of specimens	1	1	–	1	2	1
Body length	855	1400	–	2022	7869	2600
Body width	253	400	–	477	1847	850
Forebody	399	–	–	983 × 500	3765 × 1714	–
Hindbody	320	–	–	706 × 366	2750 × 959	–
Preoral lobe length	43	–	–	22	88	–
Ventral sucker	732 × 723	250*	800 × 630	349 × 321	1257 × 123	550*
Oral sucker	391 × 455	190*	500 × 450	222 × 207	655 × 708	250*
Pharynx	177 × 214	–	180 × 160	91 × 96	254 × 275	–
Sinus sac	150 × 211	–		97 × 92	289 × 291	–
Seminal vesicle	102 × 35	–	310 × 90	119 × 32	291 × 134	–
Left testis	170 × 237	–	340 × 190	115 × 88	271 × 353	–
Right testis	160 × 225	–		136 × 101	256 × 280	–
Ovary	267 × 289	–	340 × 110	129 × 107	–	–
Left vitelline mass	508 × 217	–	540 × 350	180 × 103	546 × 658	–
Right vitelline mass	483 × 312	–		204 × 109	550 × 446	–
Eggs	61 × 39	45 × 20	56 × 26	58 × 39	62 × 39	55 × 30

Abbreviations: CM, Central Mediterranean.

The presence of *Derogenes* sp. in *P. tentacularis* is not unusual as there are previous records of Derogenidae in blenniid fishes. For instance, Mediterranean blenniid host species of the related halipegine derogenid genus *Magnibursatus* Naidenova, 1969 [29, 51]. The peacock blenny *Salaria pavo* is reported as host for *M. skrjabini* (Vlasenko, 1931) [2, 29] and for *M. blennii* (Paggi & Orecchia, 1975) [29], whereas the tompot blenny *P. gattorugine*, the rusty blenny *P. sanguinolentus*, the ‘futarra’ *Paralipophrys trigloides* and *Parablennius* sp. are also hosts for *M. blennii* [29, 51]. Although we have only two specimens, the presence of an external seminal vesicle (*vs.* internal in *Magnibursatus* [20]), a uterus extending posterior to vitelline masses (*vs*. almost entirely anterior to vitellarium in *Magnibursatus* [20]), having lobed vitelline masses (*vs*. entire in *Magnibursatus* [20]) and especially the eggs lacking filaments (*vs*. present at each end in *Magnibursatus* [20]) indicate that the derogenid collected by A. Looss agrees with *Derogenes* rather than *Magnibursatus.*

Lebour [34] described some *D. varicus* adults with a similar “spiny” appearance and stated *“… curious fact noticed is that all these larval D. varicus are beset with small spines*, *whereas it is a characteristic of the adult that although it has sometimes a wrinkling of the skin*, *it is unarmed and usually smooth. It is possible that these wrinkles may be the remains of the spines fused together. The spines are especially distinct in the younger specimens*.” Interestingly, some of her specimens also contained eggs (i.e., progenetic). She also showed this spination in her figures [34]. Hence, this “*D. limula*” that we described as *Derogenes* sp., is arguably an immature *D. ruber*. We could not find any published records for *Derogenes* in Blenniidae in the Mediterranean, but *D. varicus* was recorded from the butterfly blenny *Blennius ocellaris* in the Atlantic [47]. Besides the localities being distant (Trieste, Mediterranean for “*D. limula*” from *P. tentacularis vs*. Plymouth, Atlantic for *D. varicus* of Nicoll [47]), “*D. limula*” from *P. tentacularis* can be readily distinguished from *D. varicu*s by having lobed vitelline masses. Hence, “*D. limula*” of A. Looss is clearly not *D. varicus*. Herein, we described it as *Derogenes* sp. pending further examination based on more specimens.

### *Progonus muelleri* (Levinsen, 1881) and *Derogenes varicus sensu lato*

The monotypic genus *Progonus* was erected by Looss [38] for *P. muelleri*, first described from waters off Greenland [35]. The two genera *Derogenes* and *Progonus* are very similar with the only difference being the presence of a cyclocoel in *P. muelleri vs*. blindly ending caeca in species of *Derogenes* [19, 35, 49]. We examined body sections of representatives of both genera, mainly *D. varicus s. s*. from *Limanda limanda* (Fig. 5C) and *P. muelleri* from *M. scorpius* and the two species can also be readily distinguished by the seminal vesicle, located in the anterior third of the midbody of *D. varicus s. s*. *vs*. in the posterior third of the midbody in *P. muelleri*. Additionally, the pars prostatica is far shorter in *P. muelleri* (see Fig. 5).

**Figure 9:**
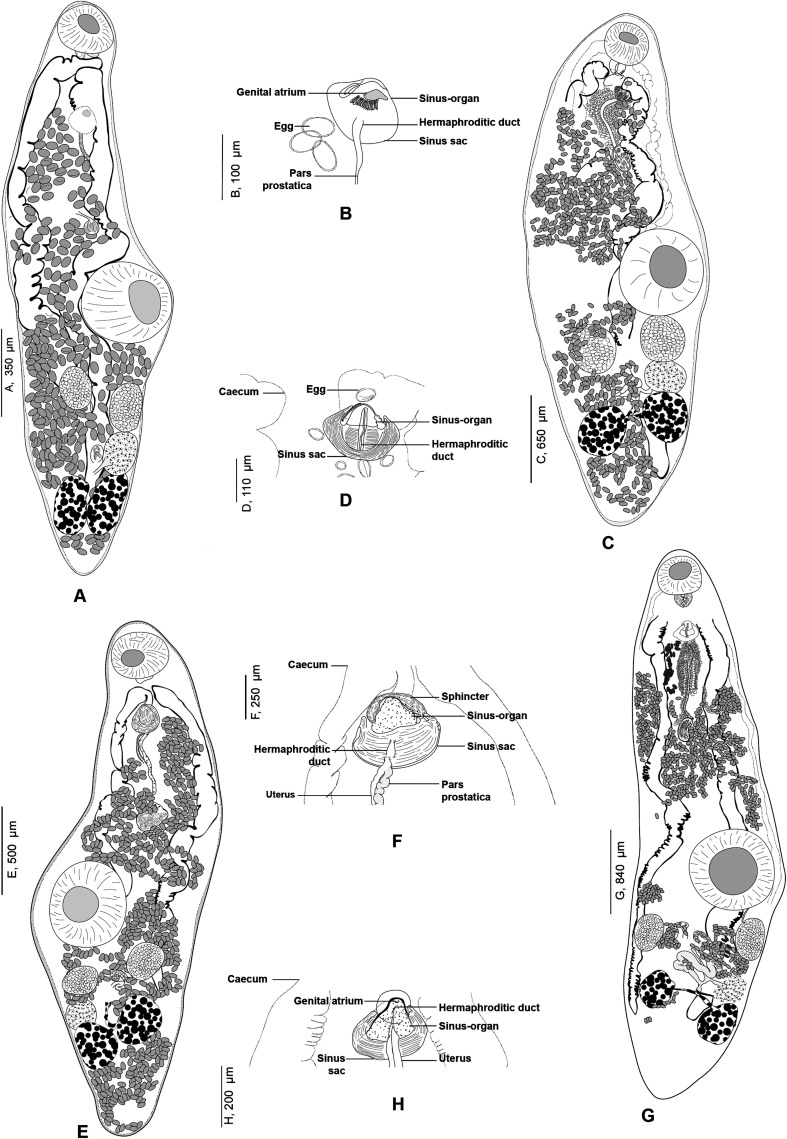
*Derogenes varicus sensu lato*. A, whole body ex *Argentina sphyraena* (SMNH-114558). B, terminal genitalia ex *A. sphyraena* (SMNH-114558). C, whole body ex *Brosme brosme* (SMNH-114560). D, terminal genitalia ex *B. brosme* (SMNH-114560). E, whole body ex *Platichthys flesus* (SMNH-208360). F, terminal genitalia ex *P. flesus* (SMNH-208360). G, whole body ex *Molva molva* (SMNH-114559). H, terminal genitalia ex *M. molva* (SMNH-114559).

**Figure 10:**
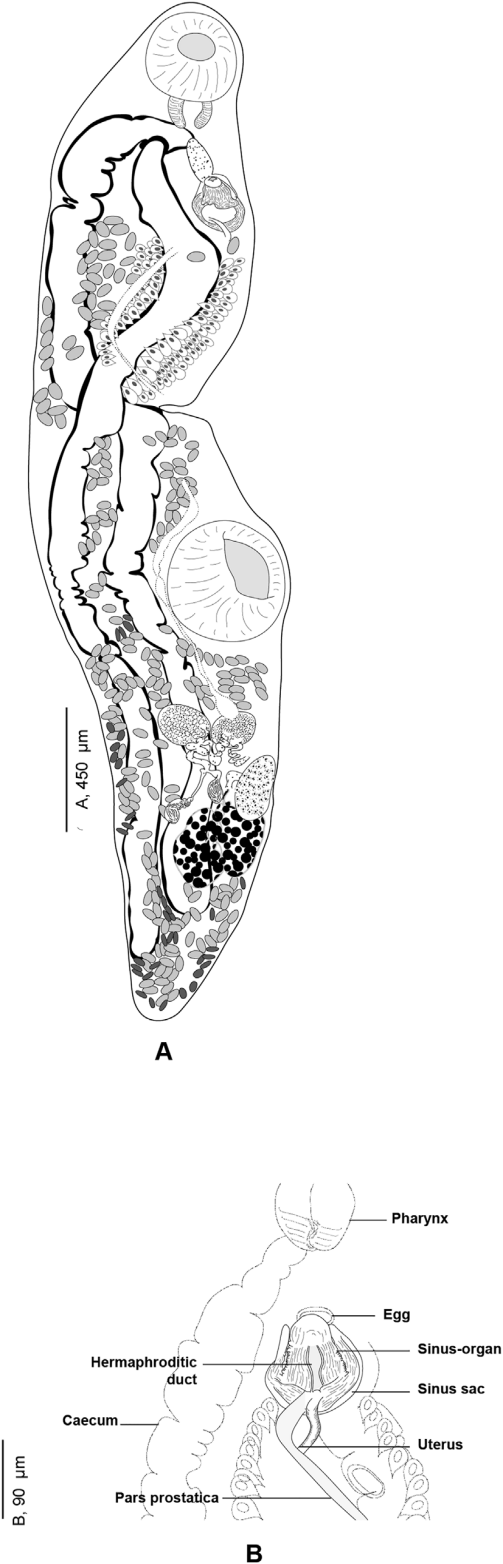
*Derogenes varicus sensu lato* ex *Hippoglossus hippoglossus* (SMNH-104577). A, whole body. B, terminal genitalia.

We examined additional specimens of *Derogenes* spp. from T. Odhner’s collections at the Invertebrates collection in the SMNH (Figs. 9 and 10), all identified and labelled as *D. varicus*, which we consider herein as representative of the *D. varicus* species complex or *D. varicus s. l*. The hosts were *Argentina sphyraena*, *Brosme brosme*, *Molva molva*, and *Platichthys flesus*. Corresponding morphometrical data are indicated in Table 7. Overall, specimens from the previously mentioned hosts share the same anatomical features, concerning the organisation of gonads and genital terminalia (Figs. 9B, 9D, 9F, 9H, 10B). *Derogenes varicus s. l*. from the previously mentioned hosts share the sausage-shaped appearance, except *D. varicus s. l.* from *B. brosme* that has a stockier appearance. The testes in *D. varicus s. l.* from *A. sphyraena* and the one from *H. hippoglossus* differ by having more longitudinally elongated testes. The most striking one is *D. varicus s. l.* from *A. sphyraena* that also differed slightly by the organisation of the genital terminalia (Fig. 9B), the shape of vitelline masses being larger and elongated, by having a smaller body and smaller organs but the measurements of eggs overlapped. However, the number of specimens measured from T. Odhner’s collection is low and thus we refrained from comparing morphometrical values. Since we are attempting to delineate species within the *D. varicus* species complex using integrative taxonomy, and as molecular sequence data are lacking for *Derogenes* from *A. sphyraena*, *B. brosme*, *M. molva*, and *P. flesus*, we consider specimens from the previously mentioned hosts as *D.* aff. *varicus* or *D. varicus s. l*., pending further examinations.

**Table 7:** Measurements of *D. varicus sensu lato* from T. Odhner’s collection examined in the present study.

Host	*Argentina sphyraena*	*Brosme brosme*	*Molva molva*	*Platichthys flesus*
Locality	Trondheim, Norway, NEA (SMNH-114558)	Trondheim, Norway, NEA (SMNH-114560)	Trondheim, Norway, NEA (SMNH-114559)	Gullmarsfjorden, Kristineberg, Sweden, NEA (SMNH-208360)
Number of specimens	1	1	1	5
Body length	–	3851	5564	2160–3369 (2756)
Body width	591	1313	1560	474–1019 (752)
Forebody	1056 × 481	1728 × 1141	2972 × 1192	1023–1559 (1273) × 444–865 (709)
Hindbody	– × 416	1521 × 938	2002 × 1198	696–1243 (1035) × 327–757 (581)
Preoral lobe length	–	73	70	28–49 (36)
Ventral sucker	367 × 334	655 × 652	839 × 848	191–561 (421) × 228–563 (431)
Oral sucker	–	323 × 357	319 × 358	243–443 (303) × 266–376 (321)
Pharynx	–	112 × 128	154 × 174	76–115 (98) × 96–110 (105)
Sinus sac	–	137 × 178	203 × 253	
Seminal vesicle	102 × 54	217 × 125	210 × 122	159–201 (180) × 82–124 (103)
Left testis	–	345 × 321	337 × 330	187–192 (190) × 210–219 (215)
Right testis	197 × 128	331 × 336	328 × 329	177–205 (191) × 155–253 (204)
Ovary	169 × 121	252 × 304	245 × 302	127–227 (183) × 112–257 (200)
Left vitelline mass	–	397 × 325	400 × 324	275–328 (304) × 200–261 (240)
Right vitelline mass	197 × 128	378 × 319	373 × 315	125–337 (266) × 115–257 (209)
Eggs	51 × 33	54 × 31	56 × 33	49–52 (50) × 30–34 (32)

Abbreviations: NEA, Northeast Atlantic.
